# Fixators dynamization for delayed union and non-union of femur and tibial fractures: a review of techniques, timing and influence factors

**DOI:** 10.1186/s13018-023-04054-3

**Published:** 2023-08-07

**Authors:** Minhua Hu, Wenxing Zeng, Jingtao Zhang, Yuanlan Feng, Luyao Ma, Feng Huang, Qunbin Cai

**Affiliations:** 1 The First Clinical College, Guangzhou University of Chinese Medicine, Guangzhou, China; 2 The First Affiliated Hospital of Guangzhou University of Chinese Medicine, Guangzhou, China

**Keywords:** Dynamization, Mechanical environment, Delayed union, Non-union, Femur and Tibial fracture, Fixators

## Abstract

The optimal balance between mechanical environment and biological factors is crucial for successful bone healing, as they synergistically affect bone development. Any imbalance between these factors can lead to impaired bone healing, resulting in delayed union or non-union. To address this bone healing disorder, clinicians have adopted a technique known as "dynamization" which involves modifying the stiffness properties of the fixator. This technique facilitates the establishment of a favorable mechanical and biological environment by changing a rigid fixator to a more flexible one that promotes bone healing. However, the dynamization of fixators is selective for certain types of non-union and can result in complications or failure to heal if applied to inappropriate non-unions. This review aims to summarize the indications for dynamization, as well as introduce a novel dynamic locking plate and various techniques for dynamization of fixators (intramedullary nails, steel plates, external fixators) in femur and tibial fractures. Additionally, Factors associated with the effectiveness of dynamization are explored in response to the variation in dynamization success rates seen in clinical studies.

**Supplementary Information:**

The online version contains supplementary material available at 10.1186/s13018-023-04054-3.

## Introduction

In recent years, the rapid socio-economic development in the healthcare sector has presented a paradox in medicine. Technological advancements have led to innovations in medical technology, facilitating the clinical application of various biomaterials and regenerative techniques that benefit patients with bone injuries. However, the increased prevalence of large vehicles has resulted in a higher incidence of trauma-related injuries, including fractures and bone defects [1, 2]. The process of bone healing is a multifaceted and intricate physiological phenomenon involving various tissues, cells, growth factors, and mechanical factors working together to regulate the osteogenic repair process [3–5]. Biomechanical factors play a significant role in bone repair, while biological factors serve as the foundation for bone healing [6]. Mechanical stimuli act as catalysts, initiating a cascade of growth factor secretion, recruitment and migration of macrophage cells, proliferation and differentiation of osteoblasts, and ultimately, completing the individual bone regeneration process [7–10].

"The concepts of "Bone adaptability" and "Cellular competition for function," originally proposed by Wolff, Roux, and Pauwel, have significantly advanced our understanding of the role of mechanical stimuli in bone development [11]. It is now established that osteoblasts are sensitive to changes in the mechanical environment [12, 13]. Different mechanical signals trigger mesenchymal stem cells (MSCs) to differentiate into three different phenotypes: fibroblasts, chondroblasts, and osteoblasts, ultimately leading to the formation of various functional tissues. For example, shear stress induces MSC differentiation into cartilage, while tensile stress leads to the formation of fibrous connective tissue, and compression stress promotes bone formation [11]. When subjected to cyclic tensile strain, bone marrow mesenchymal stem cells (BMSCs) differentiate towards osteoblasts or fibroblasts, with increasing expression of markers such as RUNX2, COL-1, OPN, SCX, COL-3, and TN-C [14, 15]. The direction of this cell differentiation correlates with the magnitude of the mechanical load. Low to moderate tensile strain conditions promote bone formation, while moderate to high loads promote fibrous formation [15]. Compression promotes the differentiation of MSCs towards chondrocytes and osteoblasts. Axial compression of medium to high amplitude induces chondrogenic gene expression, while low amplitude load induces osteogenic markers and gene expression, such as alkaline phosphatase, osteocalcin, and RUNX2, leading to the differentiation of BMSCs towards osteoblasts [15–17]. Fluid shear strain has also been shown to promote osteogenic differentiation in MSCs, as evidenced by increased expression of alkaline phosphatase and osteogenic protein-2 gene [18]. In addition to regulating cell differentiation during the repair phase, mechanical stimulation also plays a crucial role in the remodeling phase of fracture healing. It facilitates the conversion of hard bone callus into lamellar bone, leading to the restoration of the complete skeletal form and resistance to deformation [19, 20]. Moreover, interfragmentary movement (IFM) generated under appropriate strain is believed to aid in callus formation, remodeling, and revascularization [21, 22]. Modifying the mechanical environment to provide appropriate stimulation may potentially enhance the bone healing process by modulating biological factors."

Disruptions in the harmonization between mechanical loads and biological factors can lead to healing disorders characterized by delayed or prolonged healing processes. In clinical practice, it is not uncommon for such delays in bone union and instances of non-union to be attributed to alterations in the mechanical and biological environment. These alterations may include factors such as excessive periosteal stripping during surgery, disruption of nutrient vasculature, advanced age, and compromised metabolic processes, which can inhibit the bone's natural repair and reconstruction mechanisms. To address these issues, interventions frequently aim to establish a favorable biological environment for bone regeneration. These interventions can involve various approaches, such as genetic therapy, platelet-rich plasma (PRP) therapy, bone grafting, and shockwave therapy [23–27]. Conversely, delayed union and non-union may also occur due to excessive loading or the use of overly rigid fixators, which are more prevalent in lower limb fractures. The complex weight-bearing functionality and specific mechanical environment of the femur and tibia contribute to the increased likelihood of delayed union and non-union in these cases. Clinical interventions, such as dynamization of fixators, exchange of nails, and plate augmentation, can improve these bone healing disorders by adjusting the flexibility of the fixation device and optimizing mechanical stability to create a favorable biological condition [28–30]. Among these interventions, dynamization of fixators offers significant advantages in a human-centered healthcare environment due to its simplicity, minimally invasive nature, and lower cost. However, the clinical application of fixator dynamization for delayed union and non-union of lower limb fractures remains controversial, particularly regarding its effectiveness and the ideal timing for dynamization. This article reviews the principles of dynamization, indications, and factors affecting dynamization, with a focus on the techniques of fixator dynamization (intramedullary nails, steel plates, external fixators) in femur and tibial fractures. It also discusses the differences regarding the timing of dynamization."

## Principle and techniques of " Dynamization " for fracture healing

### Dynamization and Principle of " Dynamization "

The traditional concept of direct fracture healing using rigid fixators to provide absolute stability to the fragments has recently been challenged by an alternative approach called secondary healing, which has gained popularity in fracture repairs [31–34]. The term "dynamization" refers to procedures that modify biomechanical stability by changing the stiffness and mobility of the fixation device. In general, dynamization facilitates the second-stage healing process of the fracture, leading to the formation of cartilage scabs. This is achieved by transitioning the implant from a rigid to a flexible construction, allowing for less restriction of bone fragments in the fracture gap, increased IFM, and reduced interfragmentary strain (IFS). According to Stephan Perren's strain theory, an IFS between 2 and 10% is more conducive to promoting differentiation of MSCs into cartilage and woven bone formation through secondary healing, leading to rapid and robust bone healing [35]. Additionally, the mechanical stimulation generated by improved microcirculation and a more flexible construct promotes the secretion of cytokines such as transforming growth factor-β (TGFβ-1), insulin-like growth factor 1 (IGF-I), vascular endothelial growth factor (VEGF), bone morphogenetic protein 2 (BMP-2), and fibroblast growth factor 2 (FGF-2). These cytokines play an essential role in osteogenesis, blood vessel growth, and the overall repair phase [36–39].

While "dynamization" typically refers to a process that facilitates bone healing by modifying the mechanical environment, it is important to acknowledge that certain processes affecting the stability of the biomechanical milieu should not be labeled as dynamization. For instance, in lower extremity fractures, the application of limb weight and muscle forces can create an altered environment at the fracture site, resulting in relative motion between fracture fragments. As the healing process progresses, these loads intensify, leading to an increased relative motion between the fragments in the healing region. Furthermore, the approach of Distraction Osteogenesis, facilitated by the Ilizarov technique, does not align with the traditional notion of dynamization. Conceptually, dynamization involves promoting the second-stage fracture healing process by reducing the rigidity of the fixation device and facilitating axial micromotion between the fracture fragments. However, the term dynamization encompasses diverse meanings and processes depending on the specific fixation devices employed (e.g., intramedullary nails, external fixators, or locking plates). Therefore, this paper will explore dynamization techniques for various fixation devices as well as specially designed dynamization devices.

### External fixator dynamization

External fixators are widely used in treating lower limb fractures, particularly open fractures of the tibia and severe fractures with multiple traumas, with an essential role in controlling local damage. However, conventional external fixators are frequently characterized by excessive rigidity during the early stages of fracture healing, which may contribute to delayed union or non-union [40]. As the significance of the biomechanical environment in bone healing gained prominence, the appreciation for the elasticity of external fixators grew among certain proponents. In 1972, Burny et al. employed a unilateral bar connected with pins to introduce greater elasticity and reduced rigidity to the framing system, thereby facilitating the formation of healing tissue [41]. Subsequently, in 1977, De Bastiani et al. devised an orthofix dynamic axial fixator (DAF) comprising a unilateral external bar and screws, enabling enhanced flexibility through adjustments in screw tightness to diminish the stiffness of the fixation device. However, it is important to note that this approach permits axial motion while limiting rotational motion, resulting in axial loading on the fractured end and mitigating the adverse impact of shear forces on bone healing [42]. This mechanism of modifying the mechanical strain on the healing tissue by progressively altering the structural stability of the frame and by relieving the axial load to induce axial micro-movements at the fracture site is referred to as "dynamization" of the external fixation device. Nevertheless, it is crucial to highlight that axial loading in this context temporarily compresses or reduces the fracture gap at the fracture end, returning to its original state once the load is removed [43]. This specific form of dynamization is known as 'elastic dynamization'. A clinical study conducted by Bastian et al. [44] demonstrated the efficacy of external fixator dynamization in the treatment of delayed union and non-union of tibial fractures using the DAF fixation technique. Once initial bone callus formation was observed, the first stage involved loosening the central 6-angle nut to initiate the dynamic axial compression process. Out of 50 patients with delayed healing and non-healing, 47 healed within an average of 2 months after dynamic treatment, resulting in a healing rate of 94% [44]. The axial load-share ratio (LS) was then utilized to predict the safe timing for external fixator removal during the second stage. Studies have shown that when the LS of the external fixator is less than 10%, the second stage can be initiated, and the external fixator can be removed to allow for weight bearing [45, 46]. This is because the stiffness of damaged bone reaches normal cancellous bone levels at this point, and the bone callus is strong enough to bear most of the weight-bearing load, reducing the risk of re-injury upon removal of the external fixator [47].

The application and advancement of external fixation braces in the 1950s were spurred by the pioneering work of G.A. Ilizarov from the USSR, who introduced the concept of tension and stress in his eponymous Ilizarov ring external fixation brace. In recent decades, a hexapod external fixator has been developed based on the traditional Ilizarov ring external fixator. This innovative system consists of two or more interconnected rings and six adjustable telescopic struts with ball heads, designed to address mechanical challenges associated with bone non-union and related issues. [48, 49]. This annular external fixator provides a stronger shear resistance than unilateral external fixators while maintaining axial micromovement at the fracture site, thereby optimizing the mechanical environment for fracture healing [50]. Currently, the Taylor spatial frames (TSFs) are utilized as hexapod external fixation braces, often in conjunction with computer-aided navigation technology, to manage complex orthopedic conditions including limb deformities, bone defects, and fracture non-union, among others [51–54]. Similar to the dynamization process of DAF immobilizers, the dynamization procedure of TSFs involves a gradual removal of struts to decrease the stability of the structure [55]. Closure of the fracture gap returns to normal after removal of the load. Retrospective analysis by Arvesen et al. [56] of a six-legged external fixator for the treatment of hypertrophic distal tibial nonunion reported healing of 34 of 37 nonhealing tibiae, achieving a healing rate of 94% (35 of 37 tibias resulted in bony union for a final union rate of 94%). Lahoti O et al. [57] evaluated the effectiveness of TSFs in treating bony nonunion in oblique tibial fractures, achieving similar results with 11 of 12 cases recovering bone healing without any intervention. However, these studies included the upfront compression distraction process along with dynamization, making it challenging to determine whether bone healing resulted primarily from dynamization or distraction osteogenesis that stimulates bone regeneration. Moreover, no prospective randomized controlled studies have been conducted to demonstrate the true efficacy of promoting bone healing after removal of the external component.

### Intramedullary nails dynamization

In recent years, intramedullary nailing has emerged as the gold standard for treating lower limb fractures, particularly femoral and tibial fractures. Its widespread adoption is due to its low infection rate, minimal scarring, and the added advantage of early mobilization [58]. The early form of intramedullary nailing featured a static locking pattern designed to provide additional stability through nail/bone interlocking at the proximal and distal ends of the bone [59]. However, this reaming technique increased the stiffness of the bone-implant system, firmly anchoring the rigid implant to the bone fragment through interlocking screws. This rigid fixation can shield the bone from stress and strain stimuli, often leading to delayed or non-healing of the bone [60, 61]. To address these issues, Grosse and Kempf introduced the concept of "dynamization" in intramedullary nails, aiming to reduce the stress-obscuring effect by delaying the removal of a set of screws distal/proximal to the fracture, converting rigid fixation into a flexible one [62]. Subsequently, this approach evolved to include the incorporation of a longitudinal "dynamic locking hole" at the proximal end of the implant, enabling limited axial movement and rotation [63]. In 1984, Winquist et al. conducted extensive experiments that solidified the concept of "dynamization" in intramedullary nails and recommended it as an adjunctive procedure for treating nonunion and non-healing bones [64, 65]. The dynamization technique, when applied to interlocking intramedullary nails, increases IFM, promoting bone healing. By reducing stress masking, it allows unimpeded axial loads to compress and close the fracture gap. "It is important to note that, unlike the elastic dynamization observed in external fixation braces, the dynamization in the context of intramedullary nails is referred to as 'axial dynamization’. It is noteworthy that the removal of the load does not result in the restoration of the previously reduced or closed fracture gap. [66]. Several animal-based studies investigating the axial dynamization of intramedullary nails have demonstrated the beneficial effects of positive dynamization through axial motion. These benefits include fracture gap closure, fragment stabilization through surface compression, and significant advantages in fractures with distinct fracture gaps capable of supporting the fragments [67, 68].

The technique of intramedullary nail dynamization is well-established and widely used in clinical practice due to its simplicity [28, 69–76]. However, the effectiveness of intramedullary nail dynamization in treating bone healing disorders has been reported with conflicting results in various studies (Additional file 1: Table S1). For instance, Pesciallo et al. [28] conducted a retrospective analysis of 41 patients with delayed union of tibial shaft fractures who underwent intramedullary nail dynamization. They found a 92.3% healing rate after removing the locking hole screw. Similarly, Litrenta et al. [73] conducted a retrospective study involving 97 tibial shaft fractures with non-union and demonstrated a high healing rate with dynamization treatment. In contrast, other studies have reported low healing rates and ineffectiveness of dynamic fixation with intramedullary nailing in improving non-union. Vaughn et al. [70] conducted a meta-analysis that reported a healing rate of 66.4% for femoral fractures treated with intramedullary nail dynamization, which was lower than the success rate of exchange intramedullary nailing for non-union. They also found that out of 35 cases with delayed healing or non-union of lower limb fractures, only 19 cases (54%) achieved complete healing, with the remaining patients requiring bone grafting or secondary surgical interventions such as intramedullary nailing for healing [70]. The variation in healing rates across studies can be attributed to several factors, including the study design, the method of dynamization used, the type of fracture, and the size of the fracture site gap.

Traditionally, dynamization of an intramedullary nail is accomplished by removing all screws from one end of the nail. However, this approach has been observed to have a lower success rate [77]. Compared to the former approach, retaining dynamic hole screws in place during the dynamization procedure leads to better outcomes [76, 78]. By retaining some screws, the stability of the intramedullary nail is reduced but not lost, resulting in a lower risk of rotation and shortening deformities compared to removing all screws. Moreover, controlled rotational stability facilitates the onset of endochondral ossification, which is critical for successful bone healing [79]. In contrast, during secondary healing, kinetic action without rotational control can result in excessive rotation at the gap, leading to cellular rupture, cessation of healing, fibrous tissue formation, and inhibition of endochondral ossification [78, 80]. Additionally, unstable fracture types, such as long oblique, spiral, comminuted, and segmental fractures, are risk factors for poor healing outcomes after dynamization [70, 81]. Wu and Chen [72] found that the healing rate after dynamization of intramedullary nails in segmental fractures with bone non-union was only 42%, while Pan et al. [69] reported a success rate of only 61.5% for locking screws with intramedullary nails removed for closed comminuted fractures with bone non-union. This often occurs because the inherently unstable environment of complex fractures tends to disrupt the balance between mechanics and biology after locking nail removal, leading to a higher failure rate. Therefore, the stiffness of the fixator in postoperative bone non-union for unstable fractures is often modified only when the fracture ends are sufficiently stable [79, 82]. Another critical variable is the size of the fracture gap. The ability of bone repair is related to the gap size, with larger gaps leading to less bone formation and lower strength of the healing bone. Clinically, a smaller gap and adequate contact pressure in the fracture ends are critical factors in improving successful healing [83]. Experimental studies have shown that fractures with gaps smaller than 2 mm produce more cartilage and bone in the fracture gap, leading to greater stability and faster healing compared to fractures with larger gaps [84]. Conversely, larger gaps result in a reduction in periosteal callus and less bone formation in the fracture gap, leading to weaker healing bones [85]. When the gap exceeds a critical bone defect, dynamization alone is inadequate for the healing process, and bone healing may fail [86].

### Locking plate dynamization (active dynamization or temporal dynamization)

In the past, plate fixation for fractures aimed to achieve absolute stability to prevent loosening of the implant due to even minor movements. However, it has been shown that overly rigid plate construction can lead to inadequate and asymmetrical callus formation, fixator failure, and union disorders [87]. To address this issue, the distal cortical locking (DCL) technique was developed to reduce construct stiffness while maintaining strength and applied to the fixation of lower limb fractures. When the screw is securely locked in the plate hole and the distal cortex, the distal cortical locking system introduces controlled dynamization through the elastic bending of a finer diameter screw within the proximal cortical hole. Under axial stress, the dynamic locking screw of the DCL system enables micromovement between the fracture blocks by inducing overall deformation. Notably, since the proximal cortex is not fixed, the micromovement at the fracture site, both proximal and distal to the plate, exhibits parallel motion (parallel micromotion). This symmetrical micromotion promotes balanced healing of the fracture, facilitating the second-stage healing process [88, 89].

**Fig. 1:**
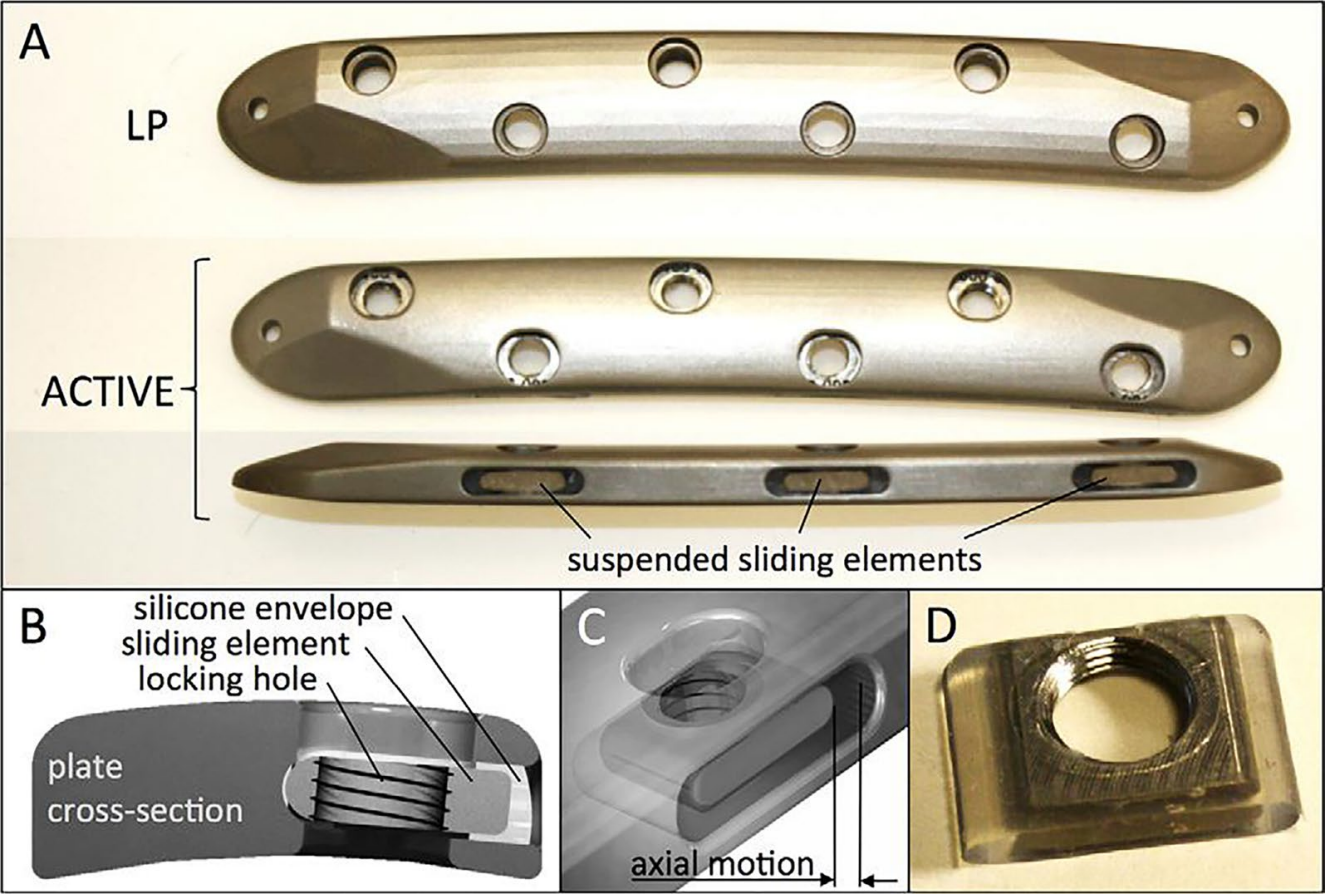
The new dynamic locking plate in which the special design of the locking holes allows axial motion of sliding elements within a certain range. **A** The new dynamic locking plate. **B**–**D**. The structure of sliding element. (Source: Bottlang et al. [91], permission by wolters kluwer health inc)

Another device for active dynamization to increase axial motion is a novel dynamic plate designed by Tsai et al. [90]. The special design of the locking holes in the plates forms the basis and core of the "active dynamization," with locking holes integrated into individual sliding elements that are elastically suspended in a silicone envelope inside lateral plate pockets. In this case, the sliding elements come with a locking hole and can move axially within a specific range, providing symmetrical axial strain to the near and far cortex (Fig. 1). The effectiveness of these plates in promoting bone healing was subsequently evaluated in an animal study by Bottlang et al. [91]. They observed homogeneous annular callus formation around the sheep tibias after plating fixation, restoring 81% strength compared to the unbroken tibias, demonstrating that the novel locking plates provide faster, stronger, and more symmetrical bone healing. Although there is a lack of evidence demonstrating the reduction of delayed healing and non-union, active dynamization creates a favorable mechanical environment for fracture healing from the initiation of fixation and has the potential to prevent non-union in the initial healing phase.

If, unfortunately, delayed healing and non-union occur during the healing process, is there a temporal dynamization of plating to reverse the outcome of these bone disorders? Drawing inspiration from the dynamization of intramedullary nails, Xu et al. [92] proposed the locking compression plate (LCP) dynamization method for the treatment of delayed bone healing and non-union in long bone fractures of the lower extremity. The LCP dynamization approach involves removing the locking screws at the more stable fracture end and replacing them with regular screws, resulting in an improved bone healing process with an 85.71% success rate and shorter healing time after plating dynamization. Similarly, Sun's [93] animal experiment yielded comparable results. The LCP dynamization procedure modifies rigid locking compression plates into more flexible constructs, reducing strain on the plate and increasing axial strain on the fracture ends. In fact, the modification of the plate-screw interface at the diaphysis level provides axial micromovement without shear or fixation failure and promotes a more uniform annular callus [93–95]. However, LCP dynamization has clinical limitations, including an increased risk of infection due to re-incision to expose the soft tissues and the potential loss of stability during screw replacement, with the risk of re-fracture of the bridging callus if the timing is inappropriate. Therefore, LCP dynamization requires a skilled surgeon to perform.

## Indications for dynamization

The classification system proposed by Weber and Cech [96] has been widely adopted to categorize bone non-unions, dividing them into atrophic and hypertrophic types. Aseptic bone non-unions can be classified into hypertrophic and atrophic non-unions based on the radiographic manifestations of callus [96]. Hypertrophic non-unions are characterized by hypervascular and vital discontinuity, associated with an overly flexible fixator or premature weight bearing, resulting in pediform or horseshoe-shaped callus on radiographs. Atrophic non-unions, on the other hand, are described by the lack of callus formation and atrophic fracture ends. They were previously considered avascular non-unions [97, 98], but current evidence suggests they have a normal vascular supply, similar to hypertrophic dysplasia [99–101]. The mechanism by which atrophic dysplasia occurs remains unclear, but some researchers suggest it is related to the reduced osteogenic capacity of mesenchymal stem cells in the fracture space, rendering them quiescent [99, 102]. Thus, atrophic non-union is more appropriately referred to as biological non-reactive, non-viable non-union. This classification is essential for both basic research and clinical practice. Several studies suggest that dynamization is more suitable for stable atrophic non-unions [82, 103], where the original fracture is primarily axially stable, such as in transverse or short oblique fractures. In such cases, a stable environment ensures that the axial load on the bone is within a safe range after the implant stiffness changes [82, 98]. Mechanical stimulation promotes local angiogenesis and osteogenesis in the non-union, resulting in eventual successful healing. In contrast, hypertrophic non-unions are characterized by biomechanical instability [97], and dynamization of fixators is equally effective in treating this condition, as stability is enhanced due to bone callus in the fracture gap, and poor alignment is less likely to occur after healing [82, 104]. However, it has also been suggested that fixator dynamization may not be necessary for hypertrophic non-unions [82].

On the other hand, unstable atrophic non-unions are generally unsuitable for flexible orthopaedic fixators [69]. Fixators with lower stiffness can cause misalignment of fracture ends, and this risk is amplified by axial instability and sparse bone callus. Complications such as limb shortening or rotational deformity may occur in such cases [70, 77, 105, 106]. Therefore, bone grafting and plate augmentation are often preferred for treating unstable atrophic non-unions, as they improve mechanical stability and the biological environment [82, 103, 107–110].

Additionally, the recently validated Non-Union Scoring System (NUSS) is an effective scoring system for non-unions, categorizing them into four classes based on the severity of various risk factors [111, 112]. The NUSS score could theoretically help predict which bone disorders could benefit more from dynamization, as low NUSS scores are associated with higher success rates. A study by Stolberg et al. [71] demonstrated that NUSS scores are a relevant factor in the success of dynamization for bone non-unions. However, more clinical studies on the NUSS score and dynamization are needed to establish the effectiveness of this treatment approach for bone non-unions.

## Ideal timing for dynamization

Interestingly, dynamizing the fixator is not only a therapeutic approach to treating bone non-union but also a preventive measure against its occurrence. When fixing limb fractures, it is wise to dynamically adjust the fixators before delayed union and non-union set in, thereby reducing the incidence of these debilitating bone healing disorders. However, the optimal timing to switch from rigid to flexible fixators remains controversial. Previous studies have investigated the effects of early (1–2 weeks) and late (3–4 weeks) dynamization on bone healing, based on animal models and finite element analysis [66, 113–116]. Takeda et al. [113] investigated the impact of mechanical stimulation on bone callus using a rat tibial fracture model and discovered that early-phase (2 days) axial mechanical stimulation encouraged hematoma formation and focal accumulation of inflammatory cells during the inflammatory response phase. It also promoted intrachondral calcification during the bone callus formation phase. Similarly, Lassion et al. [114] studied the effect of early dynamization (1 week) on periosteal bone callus development, new bone formation, callus tissue formation, and mechanical strength. They performed axial dynamization with Orthofix external fixators one week postoperatively and observed greater torsional stiffness and a more uniform distribution of periosteal bone callus on the dynamized side of the tibia. However, they found less bone and cartilage volume compared to rigid fixators [66]. In contrast, Claes et al. [66] demonstrated that early dynamization (1 week) did not promote fracture healing. They investigated the impact of early axial dynamization by reducing the unilateral external fixator stiffness one week postoperatively and evaluated stiffness, callus mineralization and volume, and tibial callus density during the healing process. Their results showed poorer bone healing measures compared to the flexible fixation group, with smaller but stiffer and better mineralized callus volume in the rigid fixation group. It is worth noting that early dynamization (1–2 weeks) has been shown to promote differentiation of mesenchymal stem cells (MSC) to the cartilage phenotype and prolong the cartilage callus formation period in several studies [66, 115]. This results in more cartilage and evenly distributed callus with potentially lower quality. However, it is not necessarily a negative outcome for the final healing outcome. Furthermore, the degree of dynamization is an overlooked crucial factor due to the difficulty of accurately manipulating the stiffness properties of fixators in clinical practice. Fu et al. [116] examined the interaction between the degree of dynamization (the stiffness variability from rigid to more flexible fixation) and timing of dynamization on fracture healing using finite element analysis. Their results showed that a high degree of dynamization (DC = 0.9) significantly delays the fracture healing process, while a moderate degree of dynamization (DC = 0.7) effectively promotes the recovery of biomechanical integrity. In conclusion, the timing and degree of dynamization play significant roles in fracture healing. Early dynamization can have varying effects on bone healing, and the optimal degree of dynamization is critical to ensure successful recovery. Further research is needed to establish precise guidelines for the application of dynamization in fracture management.

Despite the valuable insights gained from animal studies, their applicability to clinical treatment is limited due to the structural and mechanical differences between animal and human skeletons. Premature modifications to the stiffness properties of fixators, especially intramedullary nails, based solely on animal study results within one to four weeks, may compromise the fixator's anti-rotation and anti-compression properties, thereby leading to suboptimal fracture healing [70, 77, 105]. Such consequences may not be acceptable in clinical practice. Although the specific outcome has not been studied, it can be reasonably anticipated. Observing healing time and healing rates in various clinical studies, a dynamization timing between 10 and 24 weeks appears to be the most suitable option [76, 78, 117]. In a retrospective analysis by Vicenti et al. [117] on patients with femoral shaft fractures who underwent dynamization at 4, 5, and 9 months after fixation, those who had screws removed after four months showed significantly shorter healing times (8.12 ± 0.95 months) compared to those dynamized at nine months (15.4 ± 1.49 months). Similarly, Zheng et al. [76] found that patients who underwent dynamization within six months of surgery exhibited higher healing rates for femoral stem fractures compared to those who underwent dynamization later. Huang et al. [78] reported that the healing rate for patients who underwent dynamization between 10 and 24 weeks after intramedullary nail placement (83.3%) was significantly higher than for those who underwent dynamization after 24 weeks (33.3%). These findings suggest that clinical dynamization during the transition from cartilaginous to hard callus is optimal for shorter bone healing times and higher healing rates. Additionally, the timing of dynamization may also be guided by the callus-to-diaphysis ratio (CDR) observed in imaging. Vaguhn et al. [70] reported a high healing rate of fractures (93%) after dynamization, as predicted by a ROC curve analysis of 24 patients with a CDR > 1.17. In another retrospective study [117], bone healing was achieved within nine months of trauma when the CDR was between 1.47 and 1.19. Although a limited number of relevant studies are available, these imaging-based evaluation indicators provide objective evidence that can be directly observed. However, further clinical studies are needed to investigate the validity of these indicators in predicting the optimal time for dynamization.

## Conclusion and future directions

This comprehensive review paper examines the various dynamization techniques and factors influencing successful bone healing in the lower limb. Different approaches such as dynamization of intramedullary nails, plates, and external fixators are available. The primary goal of dynamization is to enhance IFM and promote bone healing by transitioning the fixation device from a rigid to a more flexible state. Simultaneously, under axial load, the fracture gap undergoes compression. In the context of dynamization, external fixation devices typically restore the fracture gap once the load is removed, a process known as elastic dynamization. On the other hand, dynamization of intramedullary nails often results in the reduction or closure of the fracture gap, a phenomenon referred to as axial dynamization. Notably, the novel dynamic plate introduces 'active dynamization,' enabling accurate bone healing through homogeneous axial movements. The effectiveness of dynamization varies based on factors such as timing, type of bone non-union, fracture type, and degree of dynamization. Early dynamization proves beneficial by increasing interfragmentary motion and promoting fracture gap closure. Moreover, dynamization is particularly suitable for stable atrophic and hypertrophic non-unions. However, it is essential to note that unstable atrophic nonunion presents a risk factor for dynamization. By considering these factors carefully, healthcare professionals can make informed decisions regarding the choice of dynamization technique, ultimately leading to successful bone healing outcomes.

While the use of intramedullary nails and external fixators for dynamization is widespread, they are not always effective as standalone treatments for bone healing disorders. There are still many other methods, such as extracorporeal shockwave therapy [27] and platelet-rich plasma [23], to create favorable biological conditions for bone repair. Studies have shown that shockwave therapy combined with dynamization can increase the healing rate [71], and it may be a wise choice to combine other adjuvant therapies for bone non-union while applying dynamization in the future. The dynamization of locking plates, on the other hand, has received less attention in both clinical and animal models, and further exploration is needed to understand its biomechanics and mechanism of action on fracture healing disorders. The type and position of screws, as well as the steel plate's design, determine the sructure's mechanical properties, which can be altered by replacing screws or employing special locking screw hole designs. Innovations in this area could potentially lead to new, effective therapeutic techniques.

Additionally, the factors that influence dynamization effectiveness mentioned in this review are just the tip of the iceberg. Other factors, such as smoking, gender, age, and soft tissue injury, have also been shown to influence bone healing after dynamization [71, 97]. Although recommendations have been made for the appropriate degree of dynamization, there is a lack of relevant research data to support these recommendations, limiting their practical application. However, with the rapid advances in computerized digital technology, orthopaedic surgical robots (Ti-robots) offer a potential solution to this problem [118]. The Ti robot has been used for intraoperative navigation and positioning, error reduction, and precise movements [119, 120], and its high data storage, processing, and conduction capabilities make it possible to accurately control the degree of dynamization [121]. The biomechanical advantages of orthopaedic surgical robot-assisted surgery have already been demonstrated [122], and its powerful in vitro biomechanical testing capabilities can accurately predict the level of dynamization that is most beneficial for bone healing, guiding clinical practice [123].

### Supplementary Information

**Additional file 1. Supplementary table S1:** Summary of intramedullary nail dynamization.

## Data Availability

Not applicable.

## Abbreviations

MSCs: Mesenchymal stem cells
BMSCs: Bone marrow mesenchymal stem cells
NUSS: Non-union scoring system
IFM: Interfragmentary movement
IFS: Interfragmentary strain
TGFβ-1: Transforming growth factor-β
IGF-I: Insulin-like growth factor 1
VEGF: Vascular endothelial growth factor
BMP-2: Bone morphogenetic protein 2
FGF-2: Fibroblast growth factor 2
DCL: Distal cortical locking
LCP: Locking compression plate
DAF: Orthofix dynamic axial fixator
LS: Load-share ratio
TSFs: Taylor spatial frames
CDR: Callus-to-diaphysis ratio
